# Outcomes of patients with lymph node metastasis treated with radical prostatectomy and adjuvant androgen deprivation therapy in a Chinese population: results from a cohort study

**DOI:** 10.1186/s12957-015-0597-3

**Published:** 2015-05-06

**Authors:** Xiaojian Qin, Chengtao Han, Hailiang Zhang, Bo Dai, Yao Zhu, Yijun Shen, Yiping Zhu, Guohai Shi, Dingwei Ye

**Affiliations:** Department of Urology, Fudan University Shanghai Cancer Center, No.270 Dong’an Road, Shanghai, 200032 China; Department of Oncology, Shanghai Medical College, Fudan University, Shanghai, Shanghai 200032 China

**Keywords:** Biochemical recurrence, Lymph node metastases, Pelvic lymph node dissection, Prostate cancer, Radical prostatectomy

## Abstract

**Background:**

The aim of this study is to assess the prognosis of prostate cancer (PCa) with lymph node metastases (LNM) detected in pelvic lymph node dissection (PLND) after radical prostatectomy (RP) and adjuvant androgen deprivation therapy (ADT) in a Chinese population.

**Methods:**

From June 2005 to September 2012, the medical histories of 67 Chinese PCa patients with LNM detected after RP and extended PLND were collected, and all these patients received continuous adjuvant ADT. Postoperative survival was estimated using the Kaplan-Meier method. The impact of various clinicopathological factors on outcome was analyzed using Cox proportional hazard regression models. All tests were two-sided with *P* < 0.05 considered significant.

**Results:**

Median follow-up was 46.7 months, and two patients were lost to follow-up. Five-year event-free survival for patients with positive lymph nodes was 93.0%, 83.0%, and 96.0% for local recurrence, systemic progression, and cancer death, respectively. One-year, 2-year, and 3-year biochemical recurrence (BCR)-free survival was 52%, 40%, and 22%, respectively. Postoperative BCR-free survival was 25.7 months. BCR-free survival for patients with a single LNM was longer than those with two or more LNM (median 39.1 months *vs*. median 17.2 months, *P* = 0.002). In a multivariate Cox model, only two or more LNM was a significant predictor of BCR (hazard ratio 2.6, *P* = 0.005).

**Conclusions:**

Despite low BCR-free survival, Chinese patients with LNM can benefit from RP and adjuvant ADT. Patients with low nodal metastatic burden had a favorable prognosis.

## Background

Prostate cancer (PCa) is the second most common cancer in men in the world. An estimated 1.1 million men worldwide were diagnosed with PCa in 2012, accounting for 15% of all cancers diagnosed in men. With an estimated 307,000 deaths in 2012, PCa was the fifth leading cause of death from cancer in men (6.6% of total men deaths). In China, although PCa incidence and mortality rates remain low (age-standardized incidence and mortality rates, 5.3 per 100,000 and 2.5 per 100,000, respectively in 2012), PCa is the most common and the most lethal male urogenital system cancer, as it is worldwide [1].

Patients who undergo staging scans prior to surgery and have no radiological evidence of pelvic lymphadenopathy, but are subsequently found to have involved pelvic lymph nodes at the time of pelvic lymph node dissection (PLND), have pathologically lymph node metastases (LNM) and have a favorable prognosis [2-4]. Patients with low-volume nodal metastases experience excellent survival rates, regardless of adjuvant treatment [5,6]. However, these studies were conducted in Western populations [2-6], and there is little data on the prognosis of PCa patients with LNM disease detected at the time of surgery in Chinese populations.

In such patients, Messing *et al*. from the Eastern Cooperative Oncology Group (ECOG) showed that early initiation of adjuvant androgen deprivation therapy (ADT) confers a survival benefit [7]; however, although a meta-analysis indicated benefit in prostate cancer-specific survival, no effect on overall mortality was identified for patients receiving adjuvant ADT, which resulted in a recommendation against adjuvant ADT [8]. Moreover, a cohort study carried out in the prostate-specific antigen (PSA) era found deferring immediate ADT in men with LNM after RP may not significantly compromise survival [9]. Regardless of variations among studies, adjuvant ADT is highly recommended for patients with LNM after radical prostatectomy (RP) worldwide range [10].

In this study, we assessed the prognosis in Chinese patients with LNM after RP and adjuvant ADT. Additionally, clinicopathological features were analyzed to identify predictors of disease progression.

## Methods

We reviewed the medical records of 1,164 consecutive patients who underwent RP and extended PLND between June 2005 and September 2012 at our institute. The extension of PLND included not only the external and obturator regions as well as the portions medial and lateral to the internal iliac vessels but also the common iliac lymph nodes at least up to the ureteric crossing. Frozen section analyses of dissected lymph nodes were not routinely performed. In total, 67 (5.8%) patients with LNM disease after surgery were identified and none of them received preoperative hormone therapy. Surgical procedures were performed by a single group of surgeons using standardized techniques. The 2002 primary tumor (T), regional lymph nodes (N), and distant metastases (M) (TNM for short) classification system was used for staging, and the Gleason system was used for grading. All of the included patients with LNM disease received continuous adjuvant ADT within 90 days of RP.

Postoperative assessments, including physical examination and serum PSA measurements, were done monthly for the initial 2 years and quarterly thereafter. Radiographic evaluation was done as indicated clinically. Biochemical recurrence (BCR) was defined as PSA 0.2 ng/mL or greater. Local recurrence was defined as cancer on biopsy of the prostatic bed or imaging studies without evidence of systemic recurrence. Systemic progression involved demonstrable metastatic deposits on radionuclide bone scan or on biopsies other than biopsy of the prostatic bed. Patients with BCR or local recurrence were referred to palliative radiation. Cause of death was identified from death certificates or physician correspondence. In lieu of a formal ethics committee, the principles of the Helsinki Declaration were followed. All human subjects provided written informed consent with guarantees of confidentiality.

IBM IPSS statistics, version 20.0 (IBM Corp., Armonk, NY, US) was employed for statistical analyses. Postoperative survival was estimated using the Kaplan-Meier method with patients censored at last follow-up or death if the end point of interest was not attained. Predictors included PSA at diagnosis, Gleason score after RP, pathological stage (pT), as well as surgical margin status (SM) and number of LNM. The impact of various clinicopathological factors on outcome was analyzed using Cox proportional hazard regression models. The hazard ratios (HR) and 95% confidence intervals (CI) associated with the presence and the number of LNM were estimated using the proportional hazards model. All tests were two-sided with *P* < 0.05 considered significant.

## Results and discussion

**Figure 1:**
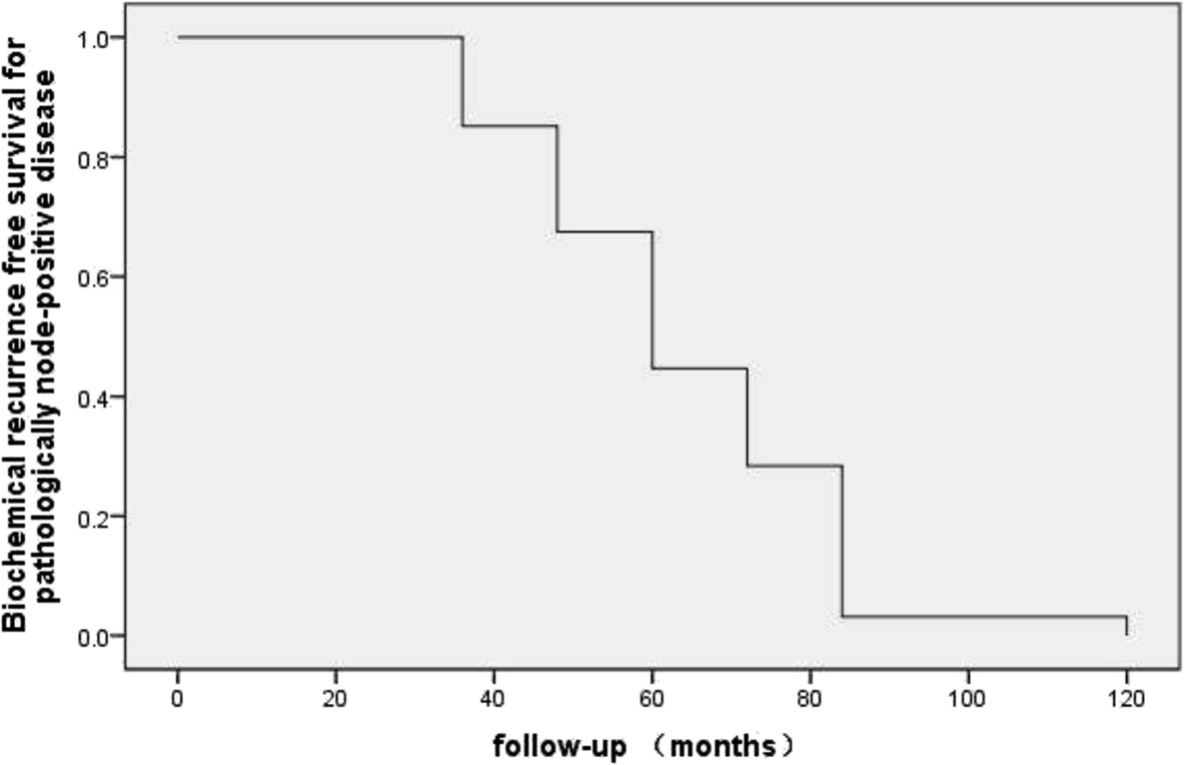
Biochemical-free survival of patients with lymph nodes metastases disease after radical prostatectomy and adjuvant androgen deprivation therapy.

The clinical characteristics of the 67 patients with LNM after surgery are summarized in Table 1. At a median follow-up of 46.7 months (range, 24 to 110 months), two patients were lost to follow-up, 43 patients had BCR, five experienced local recurrence, seven had systemic relapse, and four died, including two from PCa. Five-year event-free survival for patients with positive lymph nodes was 93.0%, 83.0%, and 96.0% for local recurrence, systemic progression, and cancer death, respectively. Since the volume of patients with local recurrence, systemic progression, and cancer death were limited, only BCR-free survival was further analyzed, and Figure 1 shows the Kaplan-Meier survival curves for patients with LNM disease. One-year, 2-year, and 3-year BCR-free survivals were 52%, 40%, and 22%, respectively.

**Table 1:** **Patient characteristics**

**Characteristics**	**Patients,** ***n*** **= 67**
Age at surgery, year ,median (IQR)	67(63~71)
Preoperative PSA, ng/ml	46.0(19.3~94.6)
Positive surgical margin, no. (%)	12(10~18)
Nodes removed, no., median (IQR)	
Positive nodes removed, no. (%)	
1	37(55.2)
2	16(23.9)
≥=3	14(20.9)
Pathologic Gleason grade, no. (%)	
≤=7	22(32.8)
>7	45(67.2)
Tumor stage, no. (%)	
≤=T2	19(28.4)
>2	48(71.6)

IQR = interquartile range; PSA = prostate-specific antigen.

**Figure 2:**
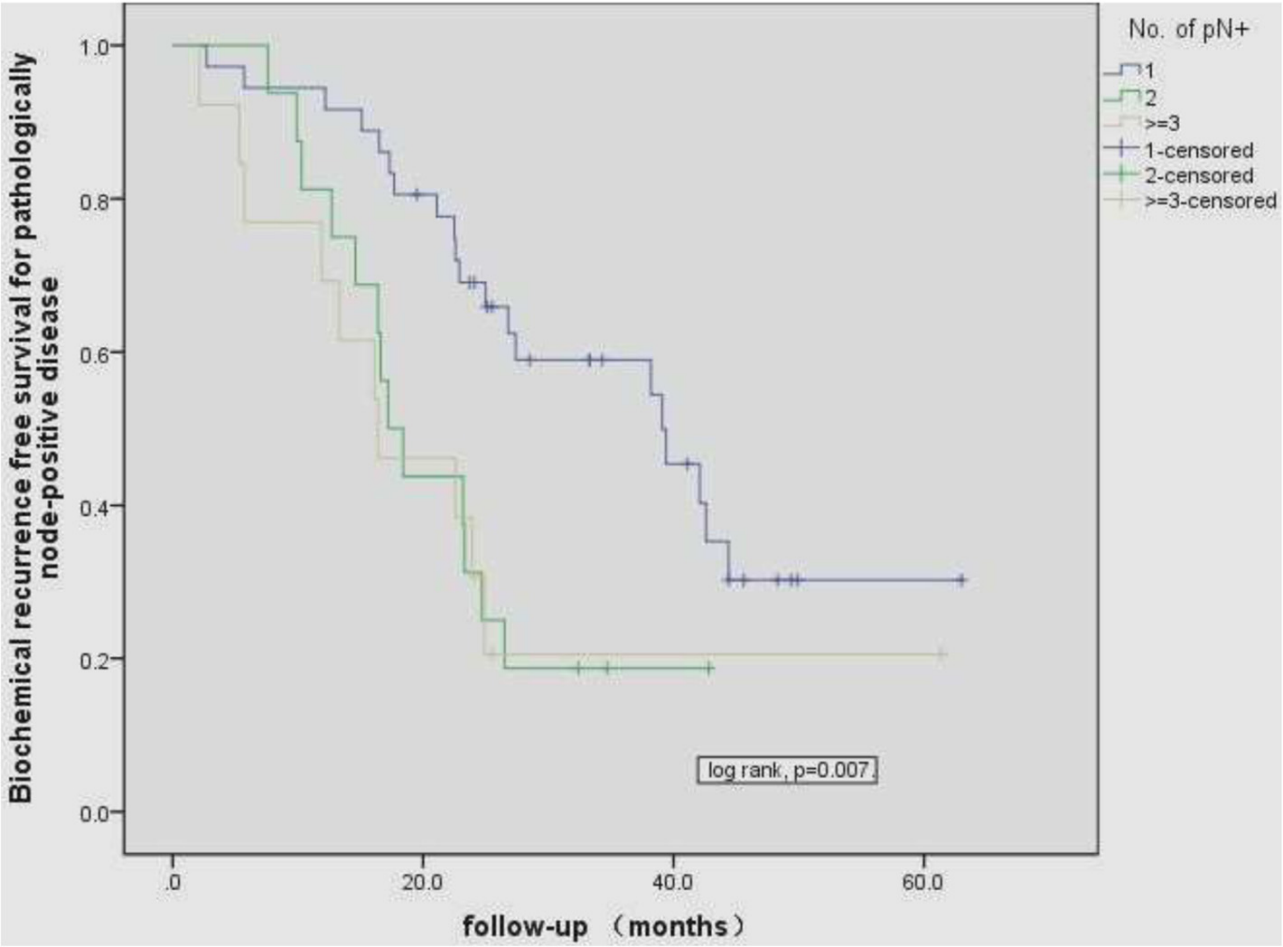
Biochemical-free survival of patients with different numbers of positive lymph nodes after radical prostatectomy and adjuvant androgen deprivation therapy.

Postoperative BCR-free survival was 25.7 months and was then stratified for all LNM patients who underwent RP by the number of positive lymph nodes (Figure 2). The BCR-free survival for patients with a single LNM was longer than that for patients with two or more LNM (median 39.1 months, 95% CI, 24.7 to 53.5, *vs*. median 17.2 months, 95% CI, 13.7 to 20.7; *P* = 0.002). Although two metastatic nodes significantly increased the risk of BCR (HR 2.7, 95% CI 1.3 to 5.5; *P* = 0.008), increasing nodal involvement did not worsen patient risk of BCR (HR 1.1, 95% CI 0.5 to 2.6; *P* = 0.797). One-year, 2-year, and 3-year BCR-free survival for patients with a single LNM and two or more LNM were 69% *vs*. 31%, 58% *vs*. 18%, and 28% *vs*. 18%, respectively.

We next examined risk factors for disease progression in patients with positive lymph nodes. In a multivariate Cox model, only two or more LNM was a significant predictor of BCR (Table 2).

**Table 2:** **Multivariate analysis of risk factors associated with biochemical recurrence from prostate cancer after radical prostatectomy in patients with node-positive disease**

**Risk factor**	**HR (95% CI)**	***P*** **value (chi-square test)**
Preoperative PSA (>=20 ng/ml or not)	0.6(0.3 to 1.4)	0.246
Pathologic Gleason grade (>7 *vs*. ≤=7)	1.1(0.6 to 2.1)	0.795
Tumor stage (pT3/4 *vs*. pT2)	1.4(0.6 to 3.0)	0.435
Positive surgical margin	0.9(0.5 to 1.9)	0.853
Positive nodes removed (2 or more *vs*. 1)	2.6(1.3 to 5.1)	0.005

HR = hazard ratio; CI = confidence interval; PSA =prostate-specific antigen.

A larger Munich Cancer Registry series reported that the incidence of nodal metastases in a European population between 1988 and 2007 was generally 10.2% during PLND and completed or abandoned RP [11]. Between the 1980s and early 2000s, nodal metastases in US patients with presumed clinically localized PCa decreased by 4% to 6%, likely because of the earlier detection of cancer with PSA screening [12-14]. The Mayo series revealed a marked decline in the incidence of positive lymph nodes at the time of RP, from 9.1% between 1988 and 1993 to 1.8% between 1998 and 2001 [2]. A small cohort of Chinese patients in Taiwan had a reported rate of LNM disease of 5.6% from 1993 to 2001 [15]. In our cohort, we experienced a similar rate (5.8%) of LNM detected after RP in Chinese patients. Presently, there is no consensus on either the treatment or prognosis in these Chinese patients.

The advent of lymph node metastasis marks the transition from localized to systemic disease. It is a generally accepted concept that disseminated malignant disease requires systemic treatment. Regardless of this paradigm, the issue of whether RP is justified in patients with limited lymph node involvement remains controversial. Because of PSA-based earlier detection of metastasis, the incidence of patients presenting with lymph node metastasis at the time of diagnosis has markedly decreased, approaching only 5% at larger institutions. Therefore, it is unlikely that a prospective randomized trial to address this issue will ever be successfully performed, thereby rendering data from carefully conducted retrospective studies important.

The Mayo series included 507 lymph node-positive patients who received RP at the time of PLND, with the longest follow-up being 10.3 years, revealed a 10-year prostate cancer-specific survival (PCSS) of 86%, with 56% of men free of BCR at the time of their last follow-up visit. In this series, 90% of the patients received adjuvant ADT and 9% received adjuvant RT. On multivariate analysis, Gleason score 8 to 10, positive surgical margins, and two or more positive lymph nodes were adverse predictors of PCSS, whereas adjuvant ADT use was not associated with systemic progression or cancer-specific survival [2]. In the Munich Cancer Registry series, 688 patients were similarly treated with RP after PLND revealed LNM disease, whereas 250 were treated without RP. About 75% of all patients received adjuvant ADT and 25% received adjuvant RT, with adjuvant therapies equally balanced between the two groups. With a median follow-up of 5.6 years, overall survival was significantly better in the RP group, and after adjustment for age, clinical T stage, number of LNM, tumor grade, and PSA level, patients who underwent RP were more likely to be alive; however, the cutoff point of two or more positive LNs reported in the Mayo series was not reproduced with their data [11].

Other series’ data have reported the prognosis of LNM disease without adjuvant ADT [3-5,16] and have come to a consensus that patients with low-volume nodal metastases experience excellent survival rates. Despite the idea that patients with LNM disease after RP have a favorable prognosis, what is not consistent among studies is how many positive nodes detected during RP are an indicator of poor prognosis [2-5,11,16]. In the present cohort with adjuvant ADT, we found the BCR-free survival for patients with a single positive node was longer than that for patients with two or more positive nodes. Although two metastatic nodes significantly increased the risk of BCR, increasing nodal involvement did not worsen the patient risk. Clinicians should be alerted to the fact that the prognosis is not good for patients with two or more positive lymph nodes after RP in Chinese patients, even with adjuvant ADT. Our data seems consistent with some previous series’ [2,5,16].

It is alarming that 1-, 2-, and 3-year BCR-free survival rates were 52%, 40%, and 22% for these patients with adjuvant ADT. Moreover, the 1-, 2-, and 3-year BCR-free survival for patients with a single positive node and two or more positive nodes were 69% *vs*. 31%, 58% *vs*. 18%, and 28 *vs*. 18%, respectively, indicating that, even for PCa patients with limited LNM, the combination of RP, extended PLND, and adjuvant ADT hardly provided a cure, and around 80% of these patients might be faced with BCR within 3 years after RP. Von Bodman *et al*. reported a 2-year BCR-free survival rate of 55% in a US cohort without adjuvant therapy [5]; with adjuvant ADT, Boorjian *et al*.’s US cohort had a 5-year BCR-free survival of 69% [2]; without adjuvant therapy, the 5-year BCR-free survival from studies by Touijer *et al*. and Bader *et al*. were 35% and 82%, respectively [4,16].

Moreover, there is no consensus on predictors of disease progression. Only specimen Gleason score and the number of positive lymph nodes were independent predictors of BCR in von Bodman’s series [5]. The same association was reproduced in a retrospective study by Touijer *et al*., which investigated the long-term outcomes of patients with LNM treated with RP without adjuvant ADT [4]. In a multivariate Cox model, only increased preoperative PSA and nondiploid tumor ploidy were significant predictors of BCR [2]; in a multivariate Cox proportional hazards model including the number of lymph node metastases, tumor stage, and Gleason score, the number of positive nodes was the only variable affecting progression and cancer-specific death [16]. In our multivariate Cox model, only two or more LNM was a significant predictor of BCR, with a hazard ratio of 2.6, compared with those with one single positive node.

We should acknowledge data of a previous Chinese cohort, in which they reported excellent prognosis following LNM disease in a single Chinese institution [17]. With laparoscopic radical prostatectomy (LRP), extended PLND (including external iliac artery, common iliac artery, obturator fossa, internal iliac, and presacral lymph nodes), and 9 months’ adjuvant ADT, 3-year BCR-free survival for their 47 LNM PCa patients was reported to be 59.2%. Additionally, they found in their cohort that there were no significant differences in BCR-free overall and cancer-specific survival rates between lymph node-positive and lymph node-negative PCa. Compared with their data, our cohort in Chinese patients failed to provide the same optimistic results.

Bader *et al*. reported that meticulous PLND with no adjuvant therapy produced intriguing results. The authors described dissection of tissues along the external iliac vein, in the obturator fossa, and along the internal iliac artery, with removal of a median of 21 lymph nodes. The projected probabilities of overall survival and PCSS for these 88 patients were both 74% at 5 years [16]. Meticulous pelvic lymph node dissection, particularly in patients with micrometastases, seems not only to be a staging procedure but may also have a positive impact on disease progression and long-term disease-free survival [16]. Compared with a median 12 lymph nodes in PLND, in the other Chinese cohort, median 19.5 lymph nodes were removed, including those from the presacral area, and this might contribute to a better prognosis in BCR-free survival [17].

However, the prognosis of Chinese patients in our cohort seemed acceptable when we considered the 5-year survival. Although the volume of patients with local recurrence, systemic progression, and cancer death were quite limited in the present cohort, the 5-year event-free survival for patients with positive lymph nodes was 93.0%, 83.0%, and 96.0% for local recurrence, systemic progression, and cancer death, respectively, which are encouraging. In a previous retrospective analysis performed in 431 consecutive patients treated for PCa at six Chinese institutions, they found that the management of PCa in China differs from that in Western countries, and the median BCR-free survival in patients with advanced diseases were 13 to 14.1 months after surgical or medical ADT [18]. Compared with their data, the prognosis of patients with LNM PCa in our cohort seemed better with a median BCR-free survival of 25.7 months. We think this study added to the body of evidence that cytoreduction of the primary tumor allows a better response in advanced PCa to androgen ablation, as was suggested by our own experience in metastatic hormone-sensitive prostate cancer (mHSPC). In our previous cohort of mHSPC patients, transurethral resection of the prostate resulted in better and more prolonged response to hormone therapy, with a trend toward positive influence in disease-specific survival and overall survival [19]. It seemed that low BCR-free survival in the present cohort was not necessarily indicative of poor prognosis in a Chinese population with LNM.

Earlier, we proposed a hypothesis that one possible mechanism that underlies the increased response to systemic therapy in advanced prostate cancer after local surgery on the primary tumor is the different androgen microenvironment inside and outside the prostate [19]. Previous investigations have found that medical castration reduces tissue androgens by 75% and also reduces the expression of several androgen-regulated genes. However, many androgen-response genes, including the androgen receptor and PSA, are not suppressed after short-term castration or after 9 months of neoadjuvant ADT. The degree of medical castration based on serum testosterone levels cannot be equated with the thoroughness of androgen ablation in the prostate microenvironment. Standard androgen deprivation does not consistently suppress androgen-dependent gene expression because of higher levels of intraprostatic androgens. Suboptimal suppression of tumoral androgen activity may lead to adaptive cellular changes, allowing prostate cancer cell survival in a low-androgen environment [20]. Because the primary tumor might be the primordial source of metastatic disease and because newly disseminated cancer cells from the castrated prostate are probably more hormone refractory, reducing the volume of intraprostatic cancer cells by local surgery before they are castration adaptive may reduce the proportion of hormone refractory cells disseminated later [21]. This idea is in accordance with what was found in the present cohort and others, as patients with low-volume nodal metastases experience better survival with RP and adjuvant ADT [2-4].

The present study has several limitations. For instance, the follow-up was not long enough to include sufficient events of local recurrence, systemic progression, or cancer death; therefore, the analysis was limited to BCR, which may restrict its immediate application for long-term survival. Although our study represents a retrospective, single center experience, the decreasing incidence of lymph node-positive prostate cancer during the PSA era makes future prospective, randomized trials of these patients unlikely. There are little data on the prognosis of PCa patients with LNM disease detected at the time of surgery in Chinese populations. As a single center cohort report, our data provide primary experience in the management of such patients, but further multicenter studies in Chinese populations are obviously needed for validation.

## Conclusions

Despite low BCR-free survival, Chinese patients with LNM can benefit from RP and adjuvant ADT. Patients with low nodal metastatic burden have a favorable prognosis. We think this study also adds to the evidence that cytoreduction of the primary tumor allows a better response of advanced PCa to androgen ablation.

## Abbreviations

ADT: androgen deprivation therapy
BCR: biochemical recurrence
CI: confidence intervals
HR: hazard ratios
LNM: lymph node metastases
PCa: prostate cancer
PLND: pelvic lymph node dissection
PSA: prostate-specific antigen
pT: pathological stage
RP: radical prostatectomy
SM: surgical margin status
TNM: primary tumor regional lymph nodes and distant metastases
